# Manipulating Adenovirus Hexon Hypervariable Loops Dictates Immune Neutralisation and Coagulation Factor X-dependent Cell Interaction *In Vitro* and *In Vivo*

**DOI:** 10.1371/journal.ppat.1004673

**Published:** 2015-02-06

**Authors:** Jiangtao Ma, Margaret R. Duffy, Lin Deng, Rachel S. Dakin, Taco Uil, Jerome Custers, Sharon M. Kelly, John H. McVey, Stuart A. Nicklin, Andrew H. Baker

**Affiliations:** 1 Institute of Cardiovascular and Medical Sciences, BHF Glasgow Cardiovascular Research Centre, University of Glasgow, Glasgow, United Kingdom; 2 Crucell Holland BV, Leiden, The Netherlands; 3 Institute of Molecular, Cell and Systems Biology, College of Medical, Veterinary and Life Sciences, University of Glasgow, Glasgow, United Kingdom; 4 University of Surrey, Guildford, Surrey, United Kingdom; Cornell University, UNITED STATES

## Abstract

Adenoviruses are common pathogens, mostly targeting ocular, gastrointestinal and respiratory cells, but in some cases infection disseminates, presenting in severe clinical outcomes. Upon dissemination and contact with blood, coagulation factor X (FX) interacts directly with the adenovirus type 5 (Ad5) hexon. FX can act as a bridge to bind heparan sulphate proteoglycans, leading to substantial Ad5 hepatocyte uptake. FX “coating” also protects the virus from host IgM and complement-mediated neutralisation. However, the contribution of FX in determining Ad liver transduction whilst simultaneously shielding the virus from immune attack remains unclear. In this study, we demonstrate that the FX protection mechanism is not conserved amongst Ad types, and identify the hexon hypervariable regions (HVR) of Ad5 as the capsid proteins targeted by this host defense pathway. Using genetic and pharmacological approaches, we manipulate Ad5 HVR interactions to interrogate the interplay between viral cell transduction and immune neutralisation. We show that FX and inhibitory serum components can co-compete and virus neutralisation is influenced by both the location and extent of modifications to the Ad5 HVRs. We engineered Ad5-derived HVRs into the rare, native non FX-binding Ad26 to create Ad26.HVR5C. This enabled the virus to interact with FX at high affinity, as quantified by surface plasmon resonance, FX-mediated cell binding and transduction assays. Concomitantly, Ad26.HVR5C was also sensitised to immune attack in the absence of FX, a direct consequence of the engineered HVRs from Ad5. In both immune competent and deficient animals, Ad26.HVR5C hepatic gene transfer was mediated by FX following intravenous delivery. This study gives mechanistic insight into the pivotal role of the Ad5 HVRs in conferring sensitivity to virus neutralisation by IgM and classical complement-mediated attack. Furthermore, through this gain-of-function approach we demonstrate the dual functionality of FX in protecting Ad26.HVR5C against innate immune factors whilst determining liver targeting.

## Author Summary

Adenoviruses are mostly considered self-limiting pathogens associated with respiratory, gastrointestinal and ocular infections; however, in immunocompromised subjects disseminated Ad infection can occur with life-threatening consequences. Many human Ads are capable of binding to coagulation factor X (FX). Following intravenous administration in animal models, FX binds directly to the major Ad capsid protein, the hexon, which subsequently results in virus accumulation in the liver. FX coating Ad5 also acts to shield against immune neutralisation via natural IgM antibodies and the classical complement system. Here we show that FX protection is not a conserved mechanism amongst Ads and identify the Ad5 hexon hypervariable regions (HVR) as the capsid proteins targeted by this host defense pathway. Furthermore, we show that genetic inclusion of Ad5 HVRs onto a native non-FX binder Ad26 to be sufficient to confer sensitivity to immune attack *in vitro* and *in vivo*. Using intravenous administration, we determine the significance of FX binding to the Ad5-derived HVRs with respect to defending the virus from neutralisation whilst mediating virus tropism. Our study gives new insight into the role of the viral HVRs and of FX at the interface between virus and host defense mechanisms.

## Introduction

For the immunocompromised host, human adenoviruses (Ad) have emerged as a significant pathogen capable of exploiting the impaired immunological response and becoming invasive, manifesting in prolonged, severe and life threatening conditions [1–5]. There are seven human species (A-G) of this common non-enveloped, double-stranded DNA virus. Whilst in healthy individuals infections are self-limiting, targeting defined tissues such as the lung, eye and gastrointestinal system over a short time frame, disseminated Ad infections occur when immunity is low (e.g. sufferers of hereditary immune deficiencies, patients undergoing immunosuppressive treatment [1–5]). Systemic infections can culminate in serious and diverse clinical syndromes, ranging from fulminant hepatic failure, coagulopathy, hemorrhagic cystitis, myocarditis, encephalopathy, nephritis to multi-organ failure [2,6]. The immunocompromised patient population is expanding due to increased use of immunosuppressive therapies (e.g. cytotoxic drugs) and consequently Ads are gaining increased recognition as a clinical problem. The presence of a high viral load in the blood is often strongly indicative of a severe outcome [5,6]. Incidence of infection is approximately 2.5–47% in stem cell transplant recipients, whilst paediatric transplantation patients are more prone to the disseminated disease, with mortality rates reaching up to 70% [5,7,8]. Despite the high risk, there is no FDA approved drug specific to treating Ad infection and therapeutic options can be limited. Advancing our knowledge of the complex mechanisms underlying Ad5 infection *in vivo* is of great importance.

Using species C Ad2/5 as the prototype, the *in vitro* infection pathway has been very well documented. Studies have finely detailed the individual steps from virus binding via the fiber knob protein to the primary cell surface coxsackie and adenovirus receptor (CAR) [9], engagement of the Ad penton base with α_v_β_3/5_ integrins leading to internalization [10] and subsequent trafficking from endosomes to nuclear import [11,12]. However, the lack of suitable animal models, which allow viral replication and closely mimic the human immune system, has challenged the study of Ad infection *in vivo*. Nevertheless an abundance of valuable information has been gained about viral spread, immune responses and methods to combat such, especially from its popularity as a viral vector for gene therapy, vaccination and virotherapy protocols. Of the 60+ human Ads identified [13], Ad5 is the virus-based gene transfer vector most frequently employed. Concomitantly, Ad5 is also one of the most seroprevalent of the family. The use of Ad5 as a viral vector has deepened our understanding of virus:host binding events, involvement of innate and adaptive immunity and of the factors leading to the substantial accumulation of Ad5 particles in the liver following bolus injection into the bloodstream. When administered intravenously (I.V.) the virus rapidly encounters a multitude of interactions with circulating blood components. These include virion neutralisation by pre-existing antibodies [14], sequestration by Kupffer cells [15], MARCO+-expressing splenic macrophages [16], polymorphonuclear leukocytes [17], natural IgM and complement opsonisation [18,19]. Binding to platelets [20,21], erythrocytes [22,23], and blood coagulation factors [24–26] all contribute to the substantial interplay between the virus and host. Dissecting the precise interactions which occur *in vivo* is key to our understanding of the virus infection pathways partnered with an individual’s defence mechanisms.

Previous work suggests that Ads belonging to species C (Ad1, Ad2 and Ad5) are more commonly associated with disseminated disease than other types and have been implicated in severe hepatic failure [5,8]. Hepatitis is a frequent and serious consequence of systemic Ad infections [27–29]. Coagulation factor X (FX) plays a fundamental role in determining the characteristic hepatic tropism of Ad5 [24–26]. Selectively blocking FX prevents Ad liver transduction in rodent and non-human primates following I.V delivery of virus [30–32]. FX binds with nanomolar affinity to the Ad5 hexon hypervariable regions (HVR), and acts as a bridge to attach the virus to *N* and *O*-linked heparan sulphate proteoglycans (HSPG) on the surface of hepatocytes [24,33,34]. Crystallographic and cryogenic electron microscopy identified contact points within and around Ad5 HVR5 and HVR7 which are responsible for interacting with the FX Gla (γ-carboxylated glutamic acid) domain [32]. Genetically swapping regions or specific amino acids within the Ad5 HVR5 and HVR7 for those of a non-FX-binding Ad (e.g. species D Ad48 or Ad26) has proven an effective strategy to abrogate the FX interaction and diminish liver transduction [32,35]. In addition to binding the FX Gla domain at the HVR7 amino acid motif T423-E424-T425, Ad5 is also capable of interacting with coagulation factor VII (FVII) at these points [36]. However, FVII does not support Ad5 transduction as it binds to the virus in an alternate orientation to FX, with dimerization of the FVII serine protease domains disguising potential HSPG receptor binding sites [36]. Further to its influence on liver transduction, Doronin *et al*. showed that FX coating the virus triggers recognition by the innate immune system via nuclear factor-κB (NFκB) activation and subsequent TLR4/TRAF6/NFκB-mediated inflammation, an effect absent when Ad5 was genetically manipulated to be devoid of FX binding [37].

Recent work by Xu *et al*. has produced additional insight into the function of FX in adenovirus biology [19]. They indicated a role for FX in protecting Ad5 from attack by natural IgM antibodies and the classical complement system upon exposure to murine blood [19]. In co-operation with IgM, complement activation acts as an innate host defense mechanism and has previously been shown to result in neutralisation of invading pathogens including Ads [18,19,38]. *In vitro* data demonstrated that FX can prevent Ad5 from this neutralisation when incubated with murine serum [19]. In contrast to studies in wild-type mice, the Ad5:FX interaction was not essential for liver transduction in mice deficient in natural antibodies or the complement components C1q and C4 [19]. Instead FX binding to Ad5 acted as a protective "shield", decorating the viral capsid and preventing natural IgM and classical complement mediated inhibition of Ad gene transfer [19]. This study has led to some speculation surrounding the impact of FX in determining Ad liver tropism.

It is evident that blood components influence Ad tropism, whilst other interactions (e.g. Kupffer cell uptake) remain dominant barriers to widespread Ad dissemination. Here, we studied the binding events and mechanisms deciding the fate of the virus in circulation. We attempted to dissect the importance of these interactions in determining viral cellular uptake and tropism. In this study we used genetically mutated Ad vectors to identify key hexon regions responsible for IgM and complement-mediated attack. The Ad5 HVRs were identified as the critical viral capsid components. We then incorporated these regions and FX binding capability onto a non-FX-binding Ad26 background. We utilised this novel vector to investigate the significance of FX and the role of the Ad5 HVRs in the interplay between viral immune recognition and tropism *in vivo*.

## Results

### Ad5 hexon hypervariable regions are required for IgM and classical complement-mediated neutralisation

**Fig 1:**
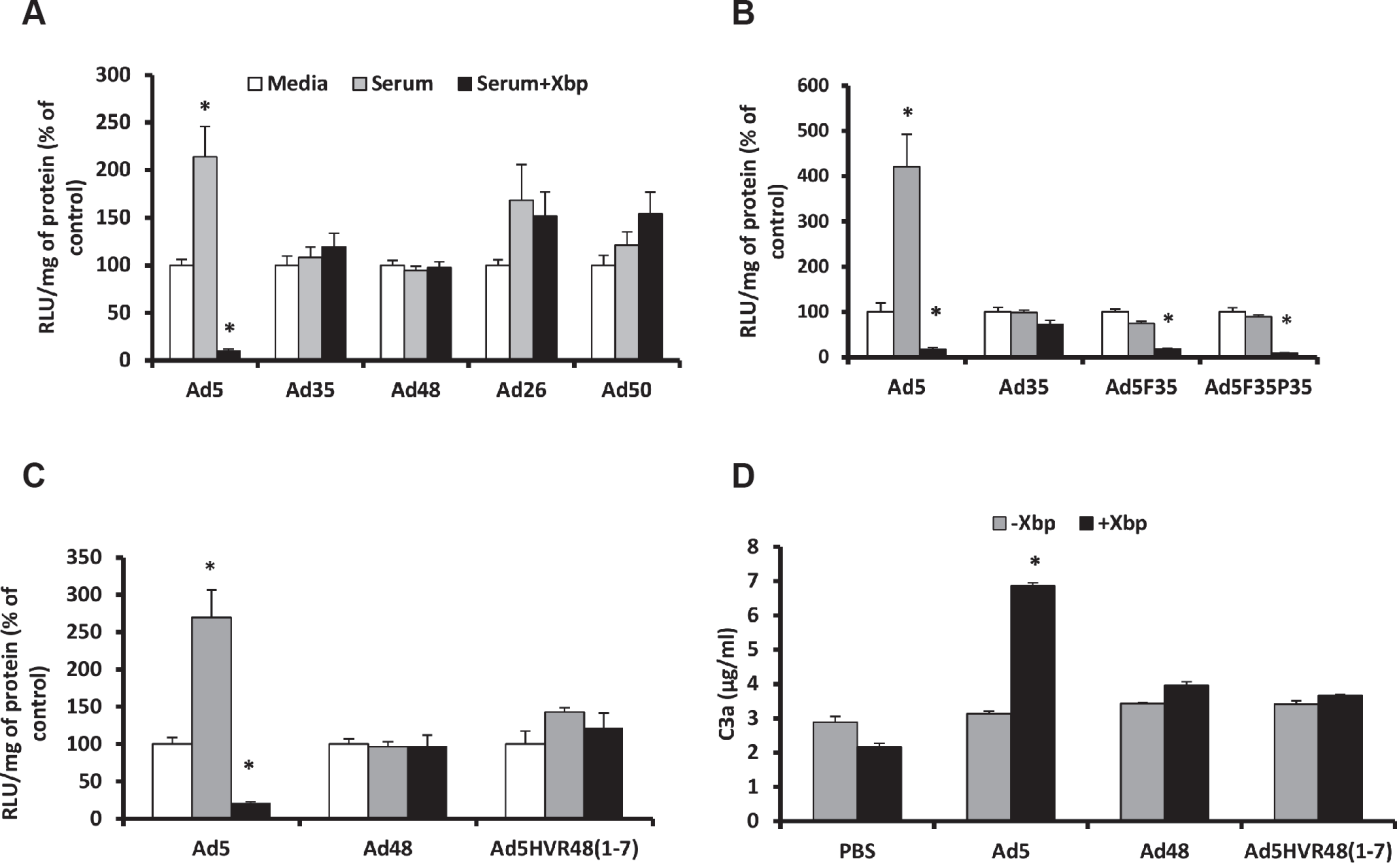
Ad5 HVRs mediate neutralisation by naïve murine serum. SKOV3 (A) or A549 cells (B-C): Vectors (2x10^10^ vp/mL) were incubated with RPMI-1640 media, 90% C57BL/6 mouse serum in RPMI-1640 media in the absence or presence of 40 μg/mL Xbp for 30 min at 37°C. Virus suspensions were diluted 200-fold in serum-free media and 100 μL added to cells for 2 h at 37°C before being replaced with RPMI-1640 media with 2% FCS. Transgene expression was quantified ∼16 h post-transduction and relative light units (RLU) normalized to mg total protein. Results for each vector are shown as percentage of the matched media control. White bars = media alone, grey bars = serum and black bars = serum + Xbp. (D) C57BL/6 mouse serum was incubated with Ad (5x10^10^ vp/mL) in the presence or absence of 40 μg/mL Xbp for 90 min at 37°C. C3a levels were quantified by ELISA. *p < 0.005 vs. matched controls.

FX coating Ad5 shields the virus against IgM and classical complement-mediated immune attack [19]. Many other Ad types also bind to human FX (hFX) [24]. However it is unknown whether they are sensitive to neutralisation via the same pathway. Therefore, we compared the sensitivity of a selected panel of Ad vectors based on different species/types; the FX-binding Ad5, Ad35 and Ad50, and non-binding Ad48 and Ad26 [24] to neutralisation by murine serum *in vitro*. When Ad5 was incubated with C57BL/6 serum there was a significant increase in transduction compared to the media control, likely due to the presence of native FX in the murine serum (Fig. 1A). However when serum was pretreated with X-bp (binds the FX Gla domain blocking Ad:FX interaction [24]) Ad5 gene transfer was dramatically reduced, to levels significantly below control conditions, consistent with previous results [19] (Fig. 1A). This demonstrates that in the absence of FX Ad5 is sensitive to neutralisation by murine serum components [19]. In contrast, Ad35, Ad50, Ad48 and Ad26-mediated cell transduction was not affected by serum regardless of the presence of X-bp (Fig. 1A), indicating that these vectors are not sensitive to the same mechanism that mediates Ad5 neutralisation. It is noteworthy that the overall charge in the region of the Ad5 hexon hypervariable loops is more negative than that of Ad26/Ad35/Ad48/Ad50 (S1 Table). Protection from neutralisation by FX is also evident for Ad5 in rat serum (S1 Fig.).

Next we investigated the role of different capsid proteins in mediating Ad5 neutralisation. For this we employed a range of Ad5-based chimeric vectors (S2 Table). We found no role for the fiber or penton base, as swapping those of Ad5 for those of Ad35 (i.e. the Ad5.F35 and Ad5.F35P35 vectors), still resulted in vector neutralisation in the absence of FX (Fig. 1B). Next we assessed the contribution of the Ad5 hexon in enabling neutralisation. Here we utilised Ad5.HVR48(1–7), which we previously showed to lack FX binding [24] (S2 Table). Ad5.HVR48(1–7) was not inhibited by serum components regardless of FX, thus illustrating an essential requirement for the Ad5 hexon HVRs in mediating neutralisation via this mechanism (Fig. 1C). In addition, unlike the parental Ad5, the Ad5.HVR48(1–7) chimeric vector caused no induction of C3 activation in serum preincubated with X-bp (Fig. 1D). Hence, Ad5.HVR48(1–7) cannot bind to FX [24] and is not sensitive to neutralisation. Therefore these data indicate a pivotal role for the Ad5 HVRs in enabling immune attack via the IgM/classical complement pathway.

### Sensitivity of Ad5 HVR mutant/FX-binding deficient viruses to neutralisation

**Fig 2:**
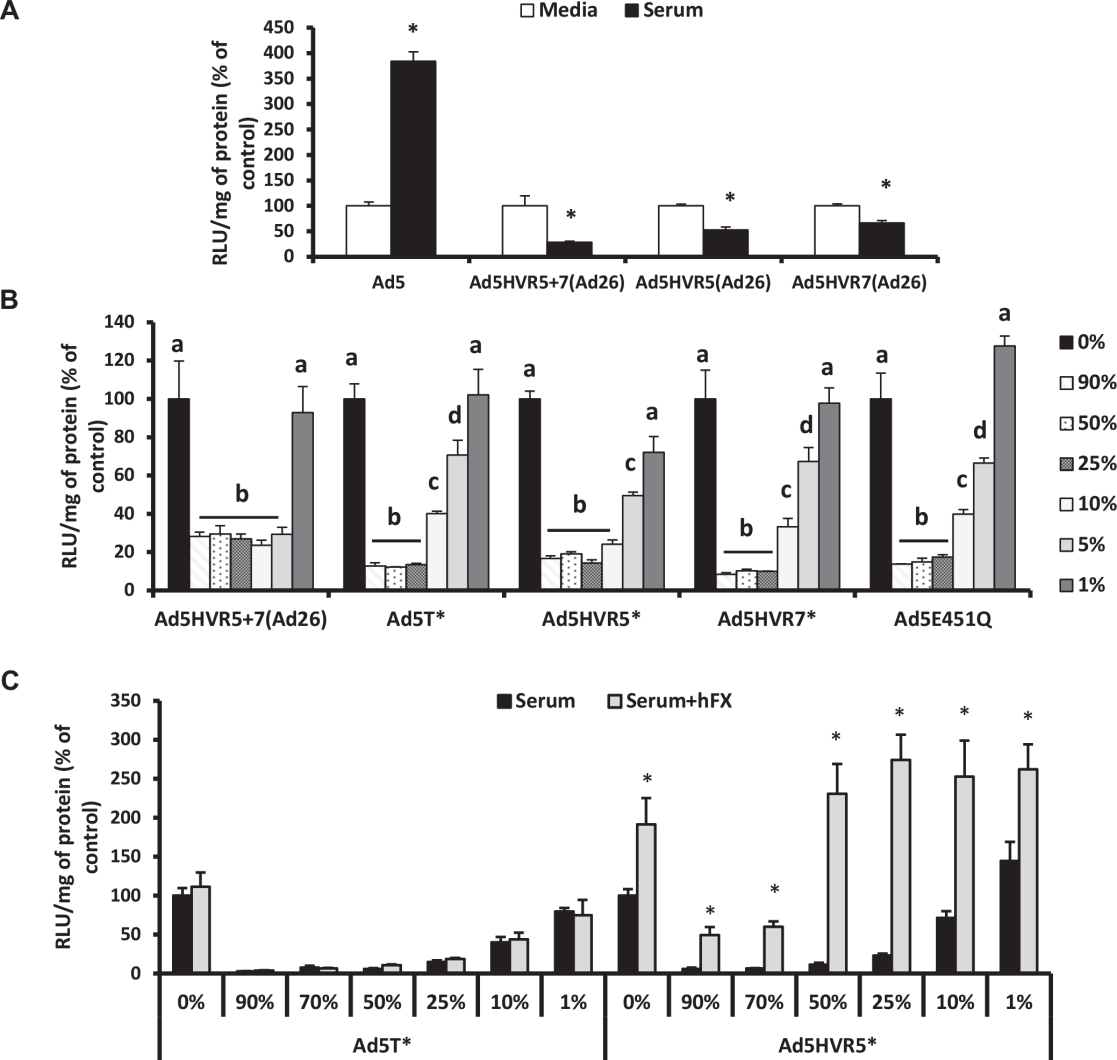
Neutralisation of FX-binding deficient Ad5 vectors by naïve murine serum. (A) A549 and (B) SKOV3 cells: Ad5 and its derivatives (2x10^10^ vp/mL) were incubated with RPMI-1640 media, 90% C57BL/6 mouse serum or 1–90% C57BL/6 mouse serum in RPMI-1640 media for 30 min at 37°C and (C) A549 cells: Ad5T* or Ad5.HVR5* (2x10^10^ vp/mL) were incubated with RPMI-1640 media, 1–90% C57BL/6 mouse serum in RPMI-1640 media in the absence or presence of 10 μg/mL hFX for 30 min at 37°C. Virus suspensions were diluted 200-fold in serum-free media and 100 μL added to cells for 2 h at 37°C before being replaced with RPMI-1640 media with 2% FCS. Transgene expression was quantified ∼16 h post-transduction and relative light units (RLU) normalized to mg total protein. Results for each vector are shown as percentage of the matched media control. Groups that do not share the same letter (a, b, c, d) are significantly different to samples within that group. *p < 0.05 vs. matched controls.

To investigate the contribution of different Ad5 HVRs in mediating neutralisation by mouse serum, we evaluated the sensitivity of Ad5 vectors containing a range of Ad26 HVR modifications. Ad26 was chosen for these studies as it does not bind FX [24] and is not susceptible to serum neutralisation *in vitro* (Fig. 1A). This series of Ad5/26 chimeric vectors (Ad5.HVR5(Ad26), Ad5.HVR7(Ad26) and Ad5.HVR5+7(Ad26)) (S2 Table) were previously shown to lack FX binding [32]. Unlike the Ad5 control, the Ad5 vectors with HVR(Ad26) swaps were all sensitive to inhibition by serum (Fig. 2A), suggesting the involvement of multiple HVRs in enabling neutralisation. Next, we employed a series of Ad5 vectors engineered with individual point mutations to alter specific amino acids to those found in Ad26 (S2 Table). These vectors were previously demonstrated to have reduced but not eliminated (e.g. Ad5.HVR5* (HVR5 mutations T270P and E271G) or abolished FX binding (e.g. Ad5.HVR7* (HVR7 mutations I421G, T423N, E424S, L426Y) and Ad5.HVR5*.HVR7*.E451Q (hereafter referred to as Ad5T*)) [32]. We evaluated vector sensitivity to neutralisation over a range of concentrations of C57BL/6 mouse serum (1–90% final volume) *in vitro* (Fig. 2B, S2 Fig.). In contrast to the media control, transduction of all FX-binding deficient Ad5 vectors was dramatically reduced in the presence of serum (Fig. 2B, S2 Fig.). This inhibitory effect was significantly lessened at lower serum concentrations (<25%) for the vectors engineered with point mutations, Ad5.E451Q, Ad5.HVR5*, Ad5.HVR7* and Ad5T* compared to vectors with entire HVR exchanges Ad5.HVR5+7(Ad26) (Fig. 2B). The latter vector remained highly sensitive to neutralisation even in the presence of 5% serum. This suggests that the sensitivity to serum is influenced by the location and extent of modifications to the Ad5 HVRs, and is, at least, partially independent of the complete loss of FX-binding capacity.

It has previously been shown that Ad5.HVR5* is capable of direct interaction with FX, albeit at lower levels than Ad5, however detailed affinity kinetics are not available [32]. We hypothesized that immune components may compete with FX for binding sites within related exposed Ad5 HVR loops. Notably we found the concentration of hFX in human serum to be ∼50% lower than that of normal plasma (S3 Table). To test whether increasing concentrations of FX could protect Ad5.HVR5* from immune attack, we spiked C57BL/6 mouse serum with 10 μg/mL hFX and examined the susceptibility of the non-FX-binding Ad5T* control vector and Ad5.HVR5* to neutralisation (Fig. 2C). As expected the presence of hFX did not affect the neutralisation of Ad5T* by serum and mediated no increase in gene transfer (Fig. 2C). However, hFX prevented neutralisation of Ad5.HVR5*, particularly in lower concentrations of serum (Fig. 2C). The presence of hFX also increased gene transfer of Ad5.HVR5* indicating its ability to both protect and act as a bridge to HSPGs despite sub-optimal FX:hexon binding conditions [24]. These data indicate that both hFX and the murine serum components can compete with one another, likely through binding to similar HVRs, and this is dependent on their relative concentrations and/or affinities.

### Generation and analysis of recombinant Ad26 virus containing modifications in hexon HVRs

**Fig 3:**
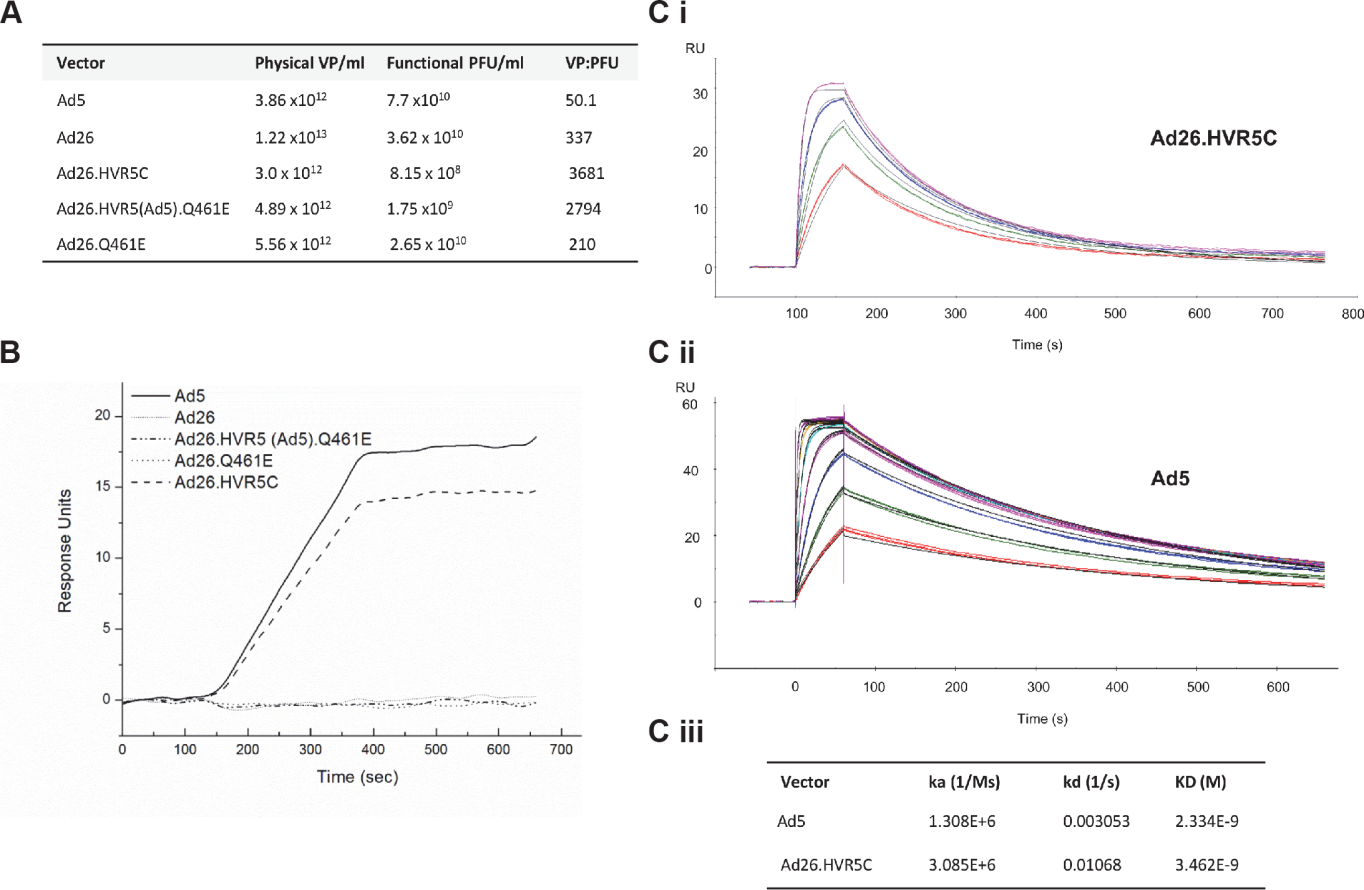
Generation of Ad26 hexon modified vectors and effects on FX binding by SPR. (A) Virus titers and vp/pfu ratios for each of the vectors produced. (B) SPR analysis of Ad5, Ad26 and mutant vector interaction with immobilized hFX (500 RU). Sensorgrams of 10^11^ vp/ml of vector injected at a flow rate of 30 μL/min. (C i) SPR analysis of hFX for immobilized Ad26.HVR5C. Samples analysed in triplicate at 4 concentrations of hFX. (C ii) SPR analysis of hFX for immobilized Ad5. Samples analysed in triplicate at 7 concentrations of hFX. The black lines represent the fit to the data. (C iii) Kinetic constants of hFX for immobilized Ad5 or Ad26.HVR5C as determined by SPR.

To further dissect the role of the Ad5 HVRs and FX in protecting Ad from serum neutralisation, we engineered FX binding capacity into Ad26 by substituting the Ad5 HVRs into the Ad26 hexon. We attempted to generate a number of Ad26-based mutants however only three Ad26 chimeras were successfully packaged into mature viral vectors at high titer (Fig. 3A, S3 Fig., S4 Table). Specific hexon sequences were chosen for mutagenesis (S3 Fig.). These included the point mutant Ad26.Q461E (the corresponding Glu residue is conserved in all FX binding Ads) and the HVR exchanges Ad26.HVR5(Ad5).Q461E and Ad26.HVR5C [in which Ad26.HVR(1–3 and 5–7) were replaced by those of Ad5], respectively (Fig. 3A, S4 Table, S3 Fig.). To note, due to the genetic capsid modifications the vp:PFU ratio was ∼10 fold higher for both Ad26.HVR5C and Ad26.HVR5(Ad5).Q461E compared to the parental Ad26 vector (Fig. 3A). We next measured the ability of each virus to bind hFX by SPR. Ad26.HVR5C showed efficient binding when injected over a hFX biosensor chip, as did the positive control Ad5, while Ad26, Ad26.Q461E and Ad26.HVR5(Ad5).Q461E did not bind hFX (Fig. 3B). We then quantified affinity kinetics (Fig. 3C). The calculated association rate constant (ka) and dissociation rate constant (kd) values of the hFX for immobilized Ad26.HVR5C were 3.085x10^6^ (1/Ms) and 1.068x10^–2^ (1/s), giving an overall equilibrium dissociation constant (KD) of 3.462x10^–9^ M. When Ad5 was immobilized and hFX was injected across the biosensor, ka and kd values were 1.308x10^6^ (1/Ms) and 3.053x10^–3^ (1/s), giving a KD of 2.334x10^–9^ M (Fig. 3C), consistent with previously reported kinetics [24]. Therefore, incorporation of Ad5 HVR(1–3 and 5–7) into Ad26 generates *de novo* binding to FX by Ad26 at an affinity similar to Ad5.

### Effect of *de novo* FX binding and Ad5 HVR inclusion on Ad26 sensitivity to mouse serum

**Fig 4:**
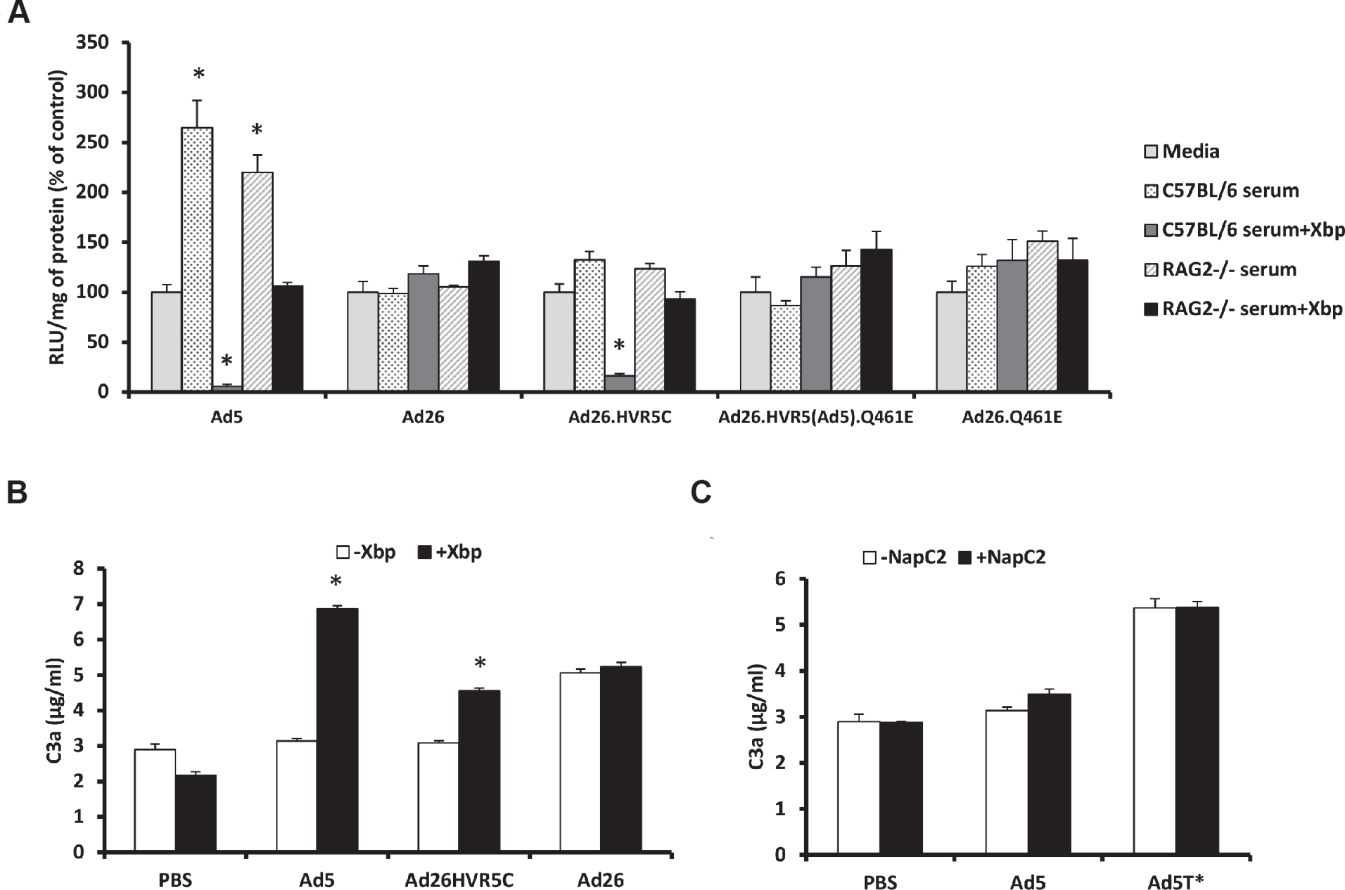
FX protects Ad26.HVR5C against immune attack. (A) 2x10^10^ vp/mL of Ad5, Ad26 or Ad26 hexon modified vectors were incubated with RPMI-1640 media, 90% C57BL/6 mouse serum, 90% C57BL/6 mouse serum with 40 μg/mL X-bp, 90% RAG^2-/-^ mouse serum or 90% RAG^2-/-^ mouse serum with X-bp, for 30 min at 37°C. Virus suspensions were diluted 200-fold in serum-free media and 100 μL added to SKOV3 cells for 2 h at 37°C before being replaced with RPMI-1640 media with 2% FCS. Transgene expression was quantified ∼16 h post-transduction and relative light units (RLU) normalized to mg total protein. Results for each vector are shown as percentage of the matched media control. *p < 0.001 vs. matched media controls. (B) C57BL/6 mouse serum was incubated with Ad (5x10^10^ vp/mL) in the presence or absence of 40 μg/mL Xbp for 90 min at 37°C. C3a levels were quantified by ELISA. (C) C57BL/6 mouse serum was incubated with Ad5 or Ad5T* (5x10^10^ vp/mL) in the presence or absence of 20 μg/mL NapC2 for 90 min at 37°C. C3a levels were quantified by ELISA. *p < 0.005 vs. matched controls.

Ad26.HVR5C provides a novel virus through which to ascertain whether inclusion of Ad5 HVR(1–3, 5–7) are sufficient for exposure to neutralisation and whether FX binding influenced the sensitivity of the parental virus to mouse serum. Therefore we assessed cellular transduction by the Ad26 mutants in the absence and presence of C57BL/6 or RAG^2-/-^ murine sera +/- X-bp. RAG^2-/-^ mice are immune-deficient, lacking mature T and B lymphocytes, closely related to the RAG^1-/-^ strain in which Ad5 liver tropism was previously demonstrated to be FX independent [19]. Parental Ad26 was resistant to neutralization by both strains of serum regardless of FX, again demonstrating Ad26 is not sensitive to the same mechanism that mediates Ad5 neutralisation (Fig. 4A). Interestingly, Ad26.HVR5C was resistant to neutralization in the presence of FX but sensitive to neutralization when immune-competent C57BL/6 serum was pre-incubated with X-bp, a similar profile to that seen with Ad5 (Fig. 4A). RAG^2-/-^ serum did not neutralize Ad26.HVR5C or Ad5 (Fig. 4A), indicating a requirement for efficient T and/or B cell antibody function [19]. Ad26.HVR5(Ad5).Q461E and Ad26.Q461E were unaffected by either sera regardless of the presence of FX. Furthermore, whilst there were differences amongst the basal levels of C3a between vectors, both Ad5 and Ad26.HVR5C, but not Ad26 enhanced C3a in a FX-dependent manner. Notably, the FX protection mechanism is not dependent on the heparin binding exosite in the FX serine protease domain, as blocking these HSPG interacting sites did not alter Ad induced C3a levels compared to the matched serum only control (Fig. 4C). Hence, inclusion of the Ad5 HVRs in Ad26.HVR5C not only leads to FX binding, but also sensitises the virus to attack in immune-competent mouse serum indicating the importance of these hexon regions in both FX binding and immune recognition.

### Effect of FX binding on recombinant Ad26 tropism

**Fig 5:**
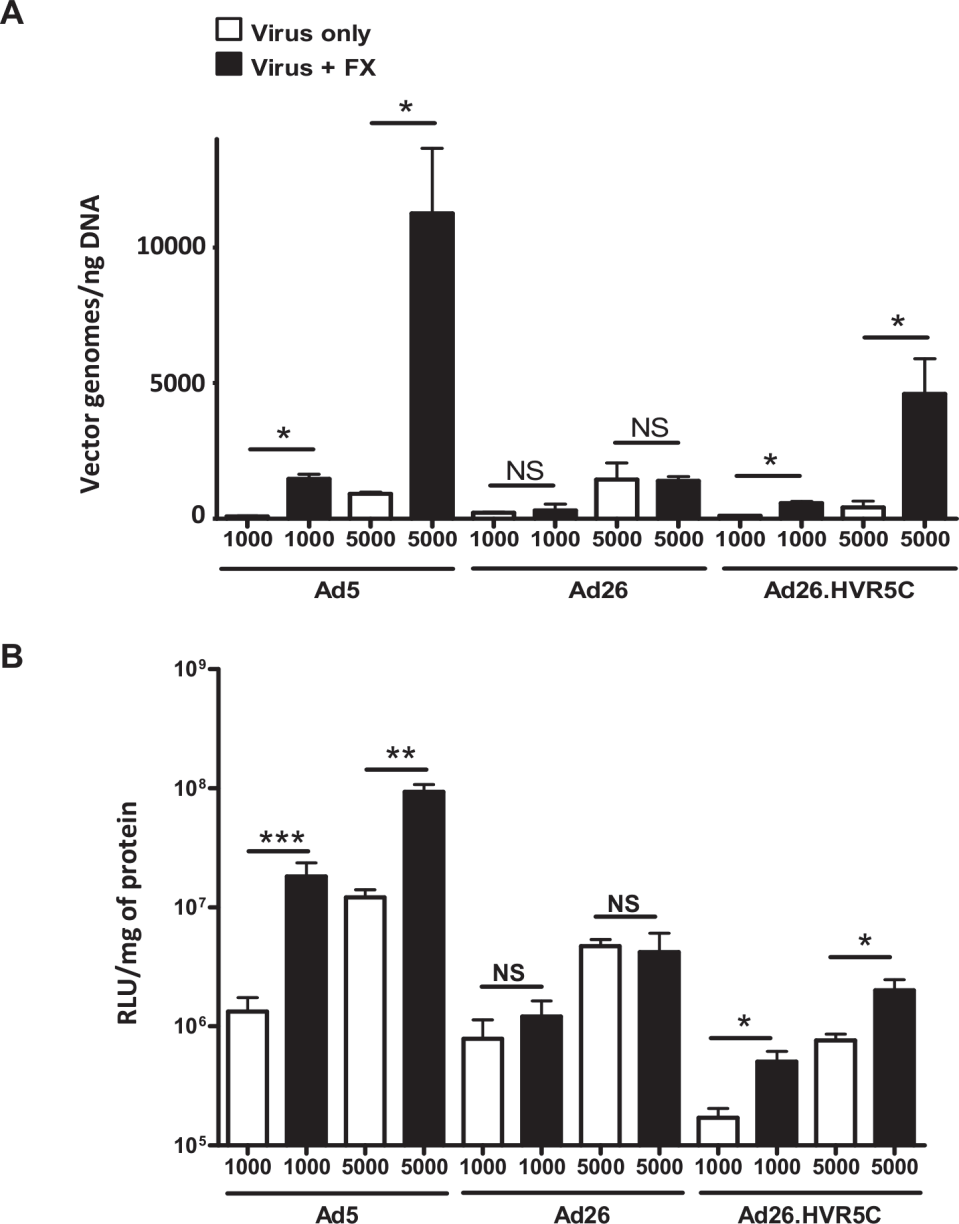
FX-mediated Ad26.HVR5C cell surface binding and transduction. (A) Binding of 1000 or 5000 vp/cell of Ad5, Ad26 or Ad26.HVR5C was quantified after incubation with SKOV3 cells for 1 h at 4°C in the presence or absence of 10 μg/mL hFX. Vector genomes were detected by quantitative PCR. (B) SKOV3 cells were infected with 1000 or 5000 vp/cell of Ad5, Ad26 or Ad26.HVR5C in the absence or presence of 10 μg/mL hFX for 3 h at 37˚C, after which media was replaced by full RPMI-1640 media and cells incubated at 37˚C. Transgene expression was measured 48 h post-infection. *p < 0.001 vs. matched -/+FX controls.

**Fig 6:**
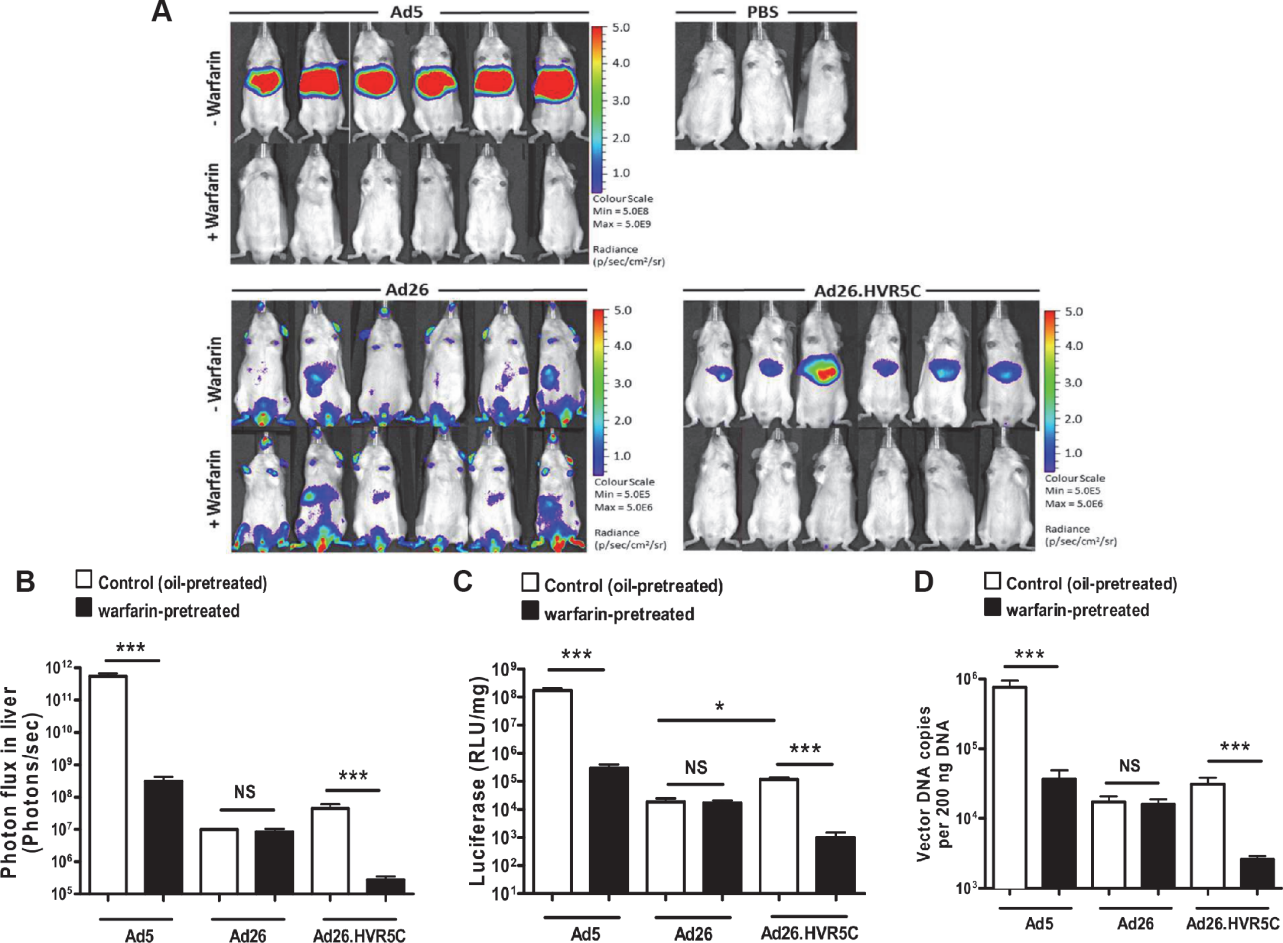
*In vivo* analysis of Ad26.HVR5C mediated liver transduction in MF1 mice. (A + B) Luciferase expression was visualised (A) and quantitatively assessed (B) by whole-body bioluminescence imaging 72 h after intravascular administration of 1x10^11^ vp/mouse Ad5, Ad26 or Ad26.HVR5C in the absence or presence of 133 μg/mouse warfarin (n = 6 animals/group) or PBS (n = 3 animals/group) in MF1 mice. (C) Livers were harvested 72 h post vector injection and processed for quantification of luciferase transgene expression and (D) quantification of viral genomes, using 200 ng of DNA for analysis. *p<0.05, ***p<0.001 and NS (not significant) vs the matched non-warfarin treated control.

We next investigated whether creating Ads engineered to bind FX influenced their ability to interact with cells *in vitro* and *in vivo*. We performed cell binding and transduction assays with Ad26.HVR5C to assess vector:cell interaction profiles (Fig. 5). SKOV3 cells were employed for these assays as they express low levels of CAR [39] and allow focus on the FX-mediated pathway. Both cell binding and transduction for Ad26.HVR5C were significantly increased in the presence of FX compared to the parental Ad26 which was unaffected by FX (Fig. 5A-B). This suggests that Ad26.HVR5C functionally binds FX leading to Ad:FX engagement with cellular HSPGs and subsequent gene transfer. Next, we examined whether the Ad26.HVR5C hexon:FX interaction generates hepatic tropism via FX bridging to hepatocytes [24,33,34]. Immune competent MF1 control mice were first treated with warfarin to deplete vitamin K-dependent coagulation factors, and then administered I.V. with 10^11^ vp of Ad5, Ad26 or Ad26.HVR5C.luc+. Luciferase expression was visualised by whole-body bioluminescence imaging and quantified at 72 h. Ad26 produced low level, widely biodistributed gene transfer and this was unaltered by warfarin (Fig. 6A). However, both Ad5 and Ad26.HVR5C produced selective transduction of the liver in non-warfarin treated mice (Fig. 6A-C) albeit the levels mediated by Ad26.HVR5C were significantly lower than those of Ad5. Despite the vp:PFU ratio of Ad26.HVR5C being ∼10 fold lower than Ad26 (Fig. 3A), there was a significant increase in liver luciferase levels for the Ad26.HVR5C vector compared to Ad26, in non-warfarin treated animals (Fig. 6C). For both Ad5 and Ad26.HVR5C, liver transduction was reduced to less than 0.5% of control following warfarin treatment. Quantification of viral genome accumulation in the livers of Ad5 and Ad26.HVR5C injected animals revealed a similar pattern of virus-mediated gene transfer (Fig. 6D). Thus, inclusion of FX binding into Ad26.HVR5C leads to profound retargeting effects following I.V. injection into MF1 mice.

### Ad5 HVRs mediate immune sensitivity *in vivo* and FX dictates Ad26.HVR5C hepatic tropism

**Fig 7:**
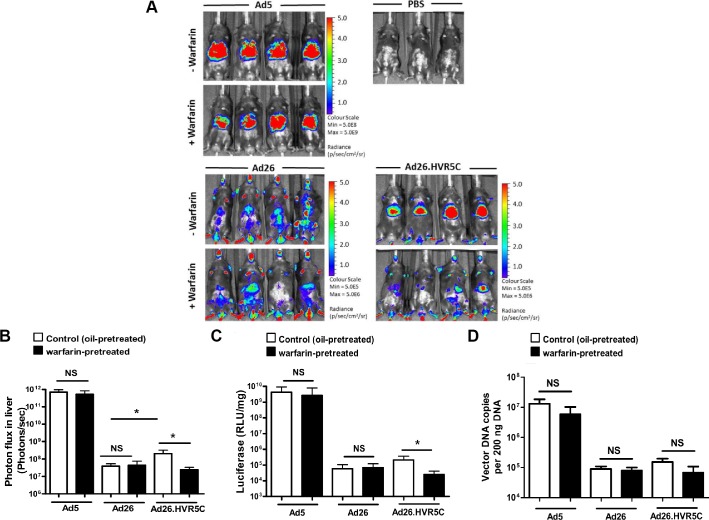
*In vivo* analysis of Ad26.HVR5C biodistribution in immune deficient mice. (A + B) Luciferase expression was visualised (A) and quantitatively assessed (B) by whole-body bioluminescence (IVIS) imaging 72 h after intravascular administration of 1x10^11^ vp/mouse Ad5, Ad26 or Ad26.HVR5C in the absence or presence of 133 μg/mouse warfarin (n = 4 animals/group) or PBS (n = 3 animals/group) in RAG^2-/-^ mice. (C) Livers were harvested 72 h post-injection and processed for quantification of luciferase transgene expression and (D) quantification of viral genomes, using 200 ng of DNA for analysis. *p<0.05 and NS (not significant) vs the matched non-warfarin treated control.

We then performed the same experiment in immune-deficient RAG^2-/-^ mice. Ad5 mediated FX-independent liver transduction in RAG^2-/-^ mice, similar to that previously reported in RAG^1-/-^ mice [19] (Fig. 7A-D). Ad26 demonstrated widespread gene expression *in vivo* which was equivalent in both non-warfarin and warfarin-treated mice (Fig. 7A). However, Ad26.HVR5C transduction was focused in the liver in non-warfarin treated RAG^2-/-^ mice, whilst hepatic gene expression was dramatically decreased in the absence of FX (Fig. 7A-C). The transduction profile of Ad26.HVR5C in warfarin-treated mice was completely altered, no longer targeting the liver but instead exhibiting a more widespread biodistribution similar to the parental Ad26 (Fig. 7A). This demonstrates the ability of the hexon:FX interaction to determine Ad26.HVR5C hepatocyte uptake. Through this gain-of-function approach, incorporation of FX binding into Ad26, FX was shown to effectively alter vector tropism *in vivo* and dictate liver targeting of Ad26.HVR5C in control and immune-deficient mice.

The high levels of gene expression by Ad5 in warfarin treated RAG^2-/-^ mice (Fig. 7A) is in contrast to the diminished luciferase expression (Fig. 6A) observed in warfarin treated MF1 mice. This is due to IgM and classical complement-mediated vector neutralisation, similar to that demonstrated previously [19]. Unlike Ad26 but in parallel to Ad5, gene expression by Ad26.HVR5C was also inhibited in warfarin treated MF1 mice but not warfarin treated RAG^2-/-^ mice (Fig. 6A, 7A). This suggests Ad26.HVR5C was neutralised in immune competent mice as a direct consequence of the engineered Ad5 HVRs. This indicates that in the immune competent setting, in the absence of a FX protective mechanism, engineering the Ad5 HVRs into Ad26 confers sensitivity to immune attack and vector neutralisation *in vivo*.

## Discussion

Here, the role of the Ad5 HVRs and the significance of FX in defending the virus from host attack whilst determining liver targeting are described. We first identified the Ad5 HVRs as responsible for conferring sensitivity to serum neutralisation. We then genetically engineered the Ad5 hexon loops, onto a non-FX-binding Ad26 background to generate the novel vector Ad26.HVR5C. Employing this as an efficient tool, we deciphered the importance of Ad5 HVRs and FX in immune recognition, protection and biodistribution following systemic Ad administration.

Previous work reported that coagulation FX coats Ad5 to protect from IgM and classical complement-mediated immune neutralisation and that Ad5 liver uptake was not solely dependent on FX under immune-deficient conditions [19]. However the capsid proteins to which IgM and the classical complement components bind and initiate this cascade eventually leading to virus inactivation have not been identified. In addition to this work, Doronin *et al*., indicated that displacement of FX from the coagulation system through binding to Ad5 is sensed by the host and an inflammatory response is initiated [37]. Therefore, with both of these recent studies in mind, we attempted to identify the Ad capsid regions responsible for mediating immune neutralisation and further decipher the functional consequences of the Ad:FX interaction. We began by showing the FX protection mechanism was not conserved amongst human Ads. Ad5 was the only type tested susceptible to serum neutralisation in the absence of FX and using a series of genetic and pharmacological approaches this was shown to be reliant on the presence of the Ad5 HVRs. We then investigated the sensitivity of Ad5-based HVR mutants and FX-deficient vectors to neutralisation by serum from immune-competent mice and found that all viruses were inhibited, but interestingly there were differences amongst these inactivation levels. These variations indicate that it is more than simply the loss of FX binding or these contact points which likely dictates immune recognition. For our experiments we used fresh mouse serum. Unlike citrated or EDTA treated plasma there is no interference with calcium, which has previously been found to influence results [18]. During the coagulation process, a proportion of the coagulation factors will be activated and levels of intrinsic FX reduced. When we investigated the effects of spiking mouse serum with hFX and compared the null-binder Ad5T* to the FX-defective binder Ad5.HVR5*, we found hFX was capable of rescuing Ad5.HVR5* from neutralisation and further increasing gene transfer to levels above the media control. These data thereby suggests that hFX and the inhibitory murine serum components compete for binding within similar regions within the Ad5 hexon. Interestingly previous data has demonstrated using targeted PEGylation that similar Ad5 HVRs (1, 2, 5 and 7) are also responsible for interacting with scavenger receptors and subsequent Kupffer cell uptake, but in that case it has been suggested that FX and the scavenger receptors do not have overlapping binding sites [40].

Our work demonstrates a key role for the Ad5 HVRs in determining virus sensitivity to serum. We successfully engineered an Ad26 vector with Ad5-derived HVRs, Ad26.HVR5C, capable of FX-mediated cell binding and transduction *in vitro*. The species D Ad26 is a less seroprevalent virus than Ad5 [14]. It has been shown to utilise both CD46 [41] and CAR [42] as primary cellular receptors and is currently gaining a lot of attention as a vector-based vaccine against HIV [43]. Interestingly, the Ad5 HVR(1–3, 5–7) exchange in this Ad26-based vector suddenly made the virus susceptible to neutralisation by mouse serum, indicating the driving influence of the HVRs and the likelihood they possess a critical IgM recognising epitope. The smaller regions of exchange in Ad26.HVR5(Ad5).Q461E or the single point mutation in Ad26.Q461E were not sufficient to induce sensitivity to neutralisation. Conversely, incorporating the Ad26 HVR5+7 into Ad5 resulted in a vector (Ad5.HVR5+7(Ad26)) that remained susceptible to the inhibitory effects of serum. This indicates it is perhaps likely that multiple HVR loops working in conjunction which are responsible for mediating the crucial serum binding event. It will require further study to fine map the essential contact sites. IgM antibodies are broadly reactive, capable of binding to unrelated structures with low affinity [44] and mostly exist as a pentameric structure, containing ten potential antigen binding sites [45]. Therefore it is possible one IgM molecule may be capable of interacting across a wide hexon region. These natural antibodies are often directed against highly repetitive and charged motifs [46,47]. A recent study suggested the involvement of the electronegative potential of antigens in IgM recognition [47]. To note, the panel of recombinant Ad5-based FX-binding deficient viruses, with the point mutations were all modulated to have lower net negative charge, whilst the more sensitive entire HVR exchanges in Ad5.HVR5+7(Ad26) had more negative charge compared to the parental vector (S1 Table). In addition it is perhaps interesting that the net charge of the Ad5 hexon protein is more negative than the other vectors tested in this study, Ad26/Ad35/Ad48/Ad50. The HVRs of Ad types tested here differ significantly and charge-specific mutation analysis is required to further address this potential issue.

Engineering FX-binding into the Ad26 vector had profound effects on viral biodistribution in *in vivo* models of viral dissemination. In complete contrast to its parental virus, Ad26.HVR5C exhibited selective liver gene transfer in the presence of FX in immune-competent and deficient mice. In the absence of FX and immunity, Ad26.HVR5C reverted to its native tropism, with the same biodistribution pattern as Ad26. This demonstrates the tropism defining force instilled by the inclusion of FX binding. The interaction with FX instils new tropism in addition to its native Ad26 receptor usage (e.g. via CAR/CD46) [41,42]. It is evident from the work presented here, and from others [19], that Ad5 remains hepatotropic in warfarin treated immune-deficient mice. Therefore this suggests, that an alternative mechanism is utilised by Ad5 to transduce hepatocytes of immune deficient animals when FX binding is diminished. Further investigation is required to answer the important question of what is the precise mechanism determining Ad5 liver targeting in the absence of FX.

The relevance of the Ad:FX interactions and protection pathways in humans suffering from the disseminated disease is yet to be examined. Further study is therefore required to investigate whether human natural IgM can bind to Ads and if this initiates similar pathways as those in the mouse i.e. classical complement mediated neutralisation in the absence of FX [19]. It may also be beneficial to directly compare and interrogate the influences and interplay of complement, natural and acquired human immunoglobulins [48]. This may help to better predict the human immune defense to widespread Ad infection or when using Ads as gene therapy vectors. In the general population the majority of specific neutralising antibodies (e.g. IgG-based) are directed against the Ad5 hexon protein and particularly focused on the Ad5 HVRs [49,50]. Previous work has demonstrated that such neutralising human sera can prevent FX-mediated cellular binding and transduction *in vitro* [51]. In addition, in MF-1 mice preinjected with human sera, higher levels of pre-existing neutralising antibodies, correlated with decreased hepatocyte transduction in the presence of FX [51]. It would be interesting to determine whether different families of human immunoglobulins (IgG/IgM/IgA) can compete for binding sites within the Ad hexon HVRs, the relative affinities of these interactions and, further, whether FX coating of the hexon affects these processes. This would also be of clinical relevance in terms of gene therapy and for determining patient suitability or the type of Ad vector employed (FX-binding or non-FX-binding vectors) for intravascular Ad administration. Investigating the relevance of the FX protection mechanism from rodent to human models is an important next step for the field. Evidently, the complexity of Ad interactions with host factors is extensive and small animal models may only provide limited information. For instance, CAR-mediated Ad5 binding to human erythrocytes is an important clinical consideration however mouse erythrocytes are devoid of CAR expression, again highlighting the necessity for using human samples [22,52,53].

Despite the high morbidity and mortality rate associated with disseminated Ad infection there are currently no licensed specific anti-adenoviral drugs available for the management of the condition. Those that have been used in clinical settings, such as ribavirin, cidofovir, and vidarabiner, have yielded varied results and treatment options remain largely unsatisfactory [54,55]. A previous study identified small molecule pharmacological agents to block FX-mediated Ad5 infection following intravenous administration in a mouse model, and found it to be an effective strategy by which to prevent hepatic targeting [56]. However, in that case the pharmacological inhibitor was acting post the Ad5:FX binding event [56]. Doronin *et al*. used cryoEM and molecular dynamics flexible fitting simulations techniques to reveal an interaction between the FX Gla K10 residue and the Ad5 HVR7 residues E424 and T425 [37]. From our work it is evident that the host natural IgM and classical complement mediated defence mechanism is effectively governed via the Ad5 HVR loops, immune recognition is dependent on more than solely the loss of FX contact points, and FX functions in mediating liver uptake in both immune competent and deficient models. Identification of a small molecule inhibitor with favourable pharmacological properties capable of specifically blocking the Ad5:FX interaction at these contact points may enable a more robust host anti-viral immune response whilst limiting liver infection and thus be very valuable in treating systemic disease.

In summary, here we have identified the Ad5 HVRs as the key regions conferring sensitivity to the IgM and complement mediated host defence pathway. FX has both a pivotal role in protecting the virus from attack and has profound effects on Ad biodistribution. Through this work we gain a greater insight into the mechanism and significance of FX in protecting Ads against innate immunity and determining virus tropism.

## Materials and Methods

### Ethics statement

All animal procedures were approved by the University of Glasgow Animal Procedures and Ethics Committee and performed under UK Home Office license PPL 60/4429 in strict accordance with UK Home Office guidelines. All efforts were made to minimize suffering.

### Cells

HEK293 (human embryonic kidney: ATCC CRL-1573) and HeLa (human cervical adenocarcinoma: ATCC CCL-2) cells were cultured in Dulbecco’s modified Eagle’s medium (DMEM; Invitrogen, Paisley, UK) and SKOV3 (human ovarian carcinoma: ATCC HTB-77) and A549 (human lung carcinoma: ATCC CCL-185) cells in RPMI-1640 medium (Invitrogen), with 2 mM L-glutamine (Invitrogen), 10% fetal calf serum (FCS; PAA Laboratories) and 1 mM sodium pyruvate (Sigma-Aldrich, UK) at 37°C 5% CO_2_. PER.C6/55K cells [57] were cultured in DMEM with 2 mM L-glutamine, 10% FCS, 1 mM sodium pyruvate and 10 mM MgCl_2_, at 37°C 10% CO_2_.

### Vector construction

E1/E3 deleted Ad5, Ad35, Ad5/35 chimeras (Ad5.F35, Ad5.F35P35), Ad26, Ad50, Ad48 and Ad5/48 chimeric (Ad5.HVR48(1–7)) vectors encoding CMV-luciferase reporter genes were generated as described previously [14,24,58]. S2 and S4 Tables provide detail on each of the mutated vectors used in this study. E1/E3 deleted Ad5 and FX-binding deficient Ad5/Ad26 chimeric vectors (Ad5.HVR5+7(Ad26), Ad5.HVR5*, Ad5HVR7*, Ad5E451Q and Ad5T*) encoding CMV-lacz reporter genes were generated as described previously [32]. Hexon gene-modified Ad26 vector genomes were constructed in the context of pAd26.luc, a plasmid that contains a PacI site-flanked, full-length Ad26 vector genome with E1, E3, and E4 deletions/modifications [14]. The vector genome is further equipped, at the site of E1 deletion, with a CMV promoter-driven expression cassette for firefly luciferase. To generate the desired hexon gene-modified pAd26.luc plasmids, the concerning Ad26/Ad5 hexon modifications (S4 Table) were first made in the context of a smaller ‘hexon shuttle’ plasmid (gene synthesis and subcloning by GeneArt/LifeTechnologies), and then shuttled into a hexon gene-deleted derivative of pAd26.luc by homologous recombination in *E*.*coli* BJ5183 (Stratagene/Agilent Technologies), as described previously [32]. S3 and S4 Figs. further describe construction and Ad26/Ad5 sequence alignment, highlighting the regions targeted for mutagenesis.

### Vector amplification

Linearised Ad plasmids were transfected in PER.C6/55K using Lipofectamine 2000 (Invitrogen). Cells were harvested 10–14 days post-transfection. Viral particles (vp) were propagated and purified by CsCl gradients. Titers were determining by protein concentrations using micro-bicinchoninic acid (BCA) Protein Assay (Thermo Scientific). Titer calculations used the formula 1 μg protein = 4x10^9^ vp and end-point dilution assays using PER.C6/55K for quantification of plaque forming units (pfu)/mL [59]. Purified Ads were analysed by SDS-PAGE followed by silver staining (Sigma-Aldrich) according to manufacturer’s instructions in order to verify the capsid composition and confirm the vector modifications did not interfere with the structural integrity of the particles.

### Surface plasmon resonance (SPR)

*Immobilized hFX*: Performed using Biacore 2000 (GE Healthcare) as described [31]. Purified hFX was purchased from Cambridge Biosciences (Cambridge, UK). hFX was covalently immobilized onto the flowcell of a CM5 biosensor chip by amine coupling. *Immobilized Ad*: Performed using T200 (GE Healthcare). Virus was biotinylated using the EZ-link sulfo-NHS-LC biotinylation kit (ThermoFisher). The biotinylated products were coupled to streptavidin-coated sensorchips (SA; Biacore); Ad5 (482RU), Ad26.HVR5C (484RU). SPR was performed in 10 mM HEPES (pH7.4) 150 mM NaCl, 5 mM CaCl_2_, 0.005% Tween20 at a flow rate of 30 μL/min and sensorchips were regenerated by injection of 10 mM HEPES (pH 7.4) 150 mM NaCL, 3 mM EDTA, 0.005% Tween20. Kinetic analysis was performed using 2-fold serial dilutions (in duplicate, starting with 30 μg/mL) of hFX and fitted using a 1:1 binding model (Biacore Evaluation software, Biacore).

### Cell binding assay

Cells were plated in 24-well formats (2x10^5^ cells/well) and incubated overnight at 37˚C. Cells were cooled (4°C) for 30 min, washed with phosphate buffered saline (PBS) before adding 1000 or 5000 vp/cell Ad -/+ hFX. hFX was used at 10 μg/ml. Cells were incubated for 1 h at 4°C, washed with PBS, and harvested. DNA was extracted from cells using the QIAamp DNA mini kit (Qiagen) and quantified using Nanodrop (ThermoScientific). Viral genomes (200 ng DNA) were quantified by quantitative polymerase chain reaction (PCR) analysis (7900HT Sequence Detection System; Applied Biosystems) using Power SYBR Green PCR mastermix and CMV primers (Applied Biosystems).

### Transduction assay

Cells were plated in 96-well formats (1x10^4^ cells/well) and incubated overnight at 37˚C. Cells were infected with 1000 or 5000 vp/cell Ad -/+ hFX. Cells were incubated for 3 h at 37°C, washed with PBS, maintained with medium and harvested 48 h post-transfection. Luciferase activity was measured using the luciferase assay (Promega, Southhampton, UK). Protein concentrations were calculated by BCA. Values expressed as relative light units (RLU)/mg of protein.

### *In vivo* transduction

8–9 week old male MF1 outbred mice (Harlan, UK) and Rag2 knockout mice (on a C57BL/6 genetic background) (kind gift from Dr Alison Michie, University of Glasgow) were used. Mice were warfarin-treated (133 μg/mouse) prior to virus administration as previously described [24]. 10^11^ vp of Ad in 100 μL PBS were administered via tail vein injection. 72 h post-injection IVIS imaging was performed and animals were sacrificed. Livers were harvested and luciferase activity measured. DNA was extracted from livers using the QIAamp DNA mini kit. Viral genomes in 200 ng DNA were quantified by qPCR as above.

### Serum neutralization assay

Cells were plated in 96-well formats (1x10^4^ cells/well) and incubated overnight at 37˚C. Fresh serum from C57BL/6 mice, Rag2^-/-^ mice or Wistar rats was separated from whole blood, diluted in RPMI-1640 and incubated with 2x10^10^ vp/mL Ad in a final volume of 50 μL. 40 μg/mL X-bp was added to samples to block FX. Where specified serum was spiked with 10 μg/mL hFX prior to Ad addition. Controls were vectors alone in serum-free medium. Vectors were incubated with serum or medium for 30 min at 37°C. Mixtures were diluted 200-fold in serum-free medium. 1000 vp/well was added for 2 h at 37°C, then replaced with medium containing 2% FBS. After ∼16 h, cells were harvested for determination of transgene expression and protein content.

### C3a ELISA

Serum was separated from fresh murine blood. Virus (5x10^10^ vp/mL) was incubated with 50 μL of serum-/+ 40 μg/mL Xbp or 20 μg/mL NapC2 (a kind gift from Dr G. Vlasuk (Corvas International, San Diego, CA, USA)) for 90 min at 37°C, then 10 mM EDTA was added. Samples were frozen at -80°C until evaluation in a mouse C3a ELISA as previously described [18]. The capture antibody for ELISA was rat anti-mouse C3a (BD Pharmingen #558250) and the detection antibody used was biotin rat anti-mouse C3a (BD Pharmingen #558251).

### FX ELISA

The levels of hFX in human serum and plasma samples were measured using a Matched-pair antibody for ELISA of human Factor X FX-EIA (Quadratech Diagnostics, Surrey, UK) according to the manufacturer’s instructions. Purified hFX (Cambridge Bioscience) was used as the control.

### Statistical analysis

Statistical significance was calculated using one-way ANOVA followed by Bonferroni *post-hoc* test with GraphPad Prism. *In vitro* results presented are representative data from three separate experiments with at least 3 experimental replicates per group. Each *in vivo* experiment was performed with a minimum of 3 animals per group (n = 4–6 for Ad treated groups, n = 3 for PBS groups). All error bars represent SEM.

## Supporting Information

S1 Fig Sensitivity to neutralisation by serum from other species. (A) Ad5 (2x10^10^ vp/mL) was incubated with RPMI-1640 media, 90% C57BL/6 mouse or Wistar rat serum in the absence or presence of 40 μg/mL X-bp for 30 min at 37°C. Virus suspensions were diluted 200-fold in serum-free media and 100 μL added to SKOV3 cells for 2 h at 37°C and then replaced with RPMI-1640 media with 2% FCS. Transgene expression was quantified ∼16 h post-transduction and relative light units (RLU) normalized to mg total protein. *p<0.001 vs. species matched media control.
(TIF)

S2 Fig Neutralization of a series of FX-binding deficient Ad5 vectors by mouse serum. (A) Ad5 and the FX-binding deficient derivatives (2x10^10^ vp/mL of each vector) were incubated with RPMI-1640 media or 90% C57BL/6 mouse serum in the absence or presence of 40 μg/mL X-bp, for 30 min at 37°C. Virus suspensions were diluted 200-fold in serum-free media and 100 μL added to SKOV3 cells for 2 h at 37°C before being replaced with RPMI-1640 media with 2% FCS. Transgene expression was quantified ∼16 h post-transduction and relative light units (RLU) normalized to mg total protein. Graphs show transduction as a percentage of control (Ad transduction with serum free media alone). *p<0.001 vs. matched the control.
(TIF)

S3 Fig Sequence alignment. Amino acid sequence alignment of the Ad5 and Ad26 hexon HVR regions to highlight domains (HVR1, HVR2, HVR3, HVR4, HVR6 and HVR7, black box; HVR5, two blue boxes) and amino acids targeted for mutagenesis studies (point mutations highlighted by *). Ad26.HVR5C = Ad26 vector in which the Ad26 HVR1–3 and 5–7 were swapped with that of Ad5.
(TIF)

S4 Fig Procedure to generate hexon-modified Ad26 vectors. (A) Hexon sequence fragments containing the desired hexon modifications were gene synthesized and subcloned, as AgeI-BamHI restriction fragments, in place of the corresponding fragment within the ‘hexon shuttle’ plasmid pHex26-Shuttle.BamHI. This plasmid carries, between two PacI sites, a 6362-bp Ad26 genomic segment (corresponding to nucleotides 15755 to 22116 of genbank accession number EF153474) encompassing the hexon coding sequence as well as left and right flanking sequences of 2 and 1.5 Kbp, respectively. The unique BamHI site present in the hexon coding sequence within this plasmid had been introduced by a silent mutation of a cytosine to thymine at a position corresponding to position 19365 in EF153474. (B) The hexon modifications were subsequently introduced into the Ad26 vector genome by homologous recombination (in E.coli) between PacI-digested hexon shuttle plasmids (containing the desired modifications) and SwaI-digested pAd26.luc.dH. This latter plasmid is a derivative of pAd26.luc that carries, between two PacI sites, an Ad26.luc vector genome whose hexon gene is replaced by SwaI sites. (C) Hexon-modified Ad26 vectors were finally rescued by digestion of the hexon-modified vector genome plasmids by PacI and transfection of the resultant digestion products into E1-complementing cells.
(TIF)

S1 Table Ad vector charge. Partial hexon amino acid sequences including the HVRs of Ad5, Ad50, Ad35, Ad26, Ad48 and chimeric vectors (see S3 Fig. for Ad5 reference sequence) were aligned. The net protein charges at pH 7.0 were calculated using CLC Genomics Workbench 6.0.5. protein analysis software.
(TIF)

S2 Table Summary of the Ad5/35/48/26 chimeric vectors. Description of the regions swapped in the chimeric vectors and their ability to bind to FX.
(TIF)

S3 Table hFX concentrations in plasma and serum. An ELISA was used to measure the concentration of FX in human plasma and serum samples.
(TIF)

S4 Table Ad26 based plasmids constructed. Summary of all Ad26-based plasmid systems constructed and the ability to rescue virus particles in PER.C6/55K cells.
(TIF)
