# Supplementary figures and images for: Manipulating Adenovirus Hexon Hypervariable Loops Dictates Immune Neutralisation and Coagulation Factor X-dependent Cell Interaction In Vitro and In Vivo
Source: PLoS Pathog. 2015 Feb 6;11(2):e1004673. doi: 10.1371/journal.ppat.1004673 (PMC4450073; doi:10.1371/journal.ppat.1004673)

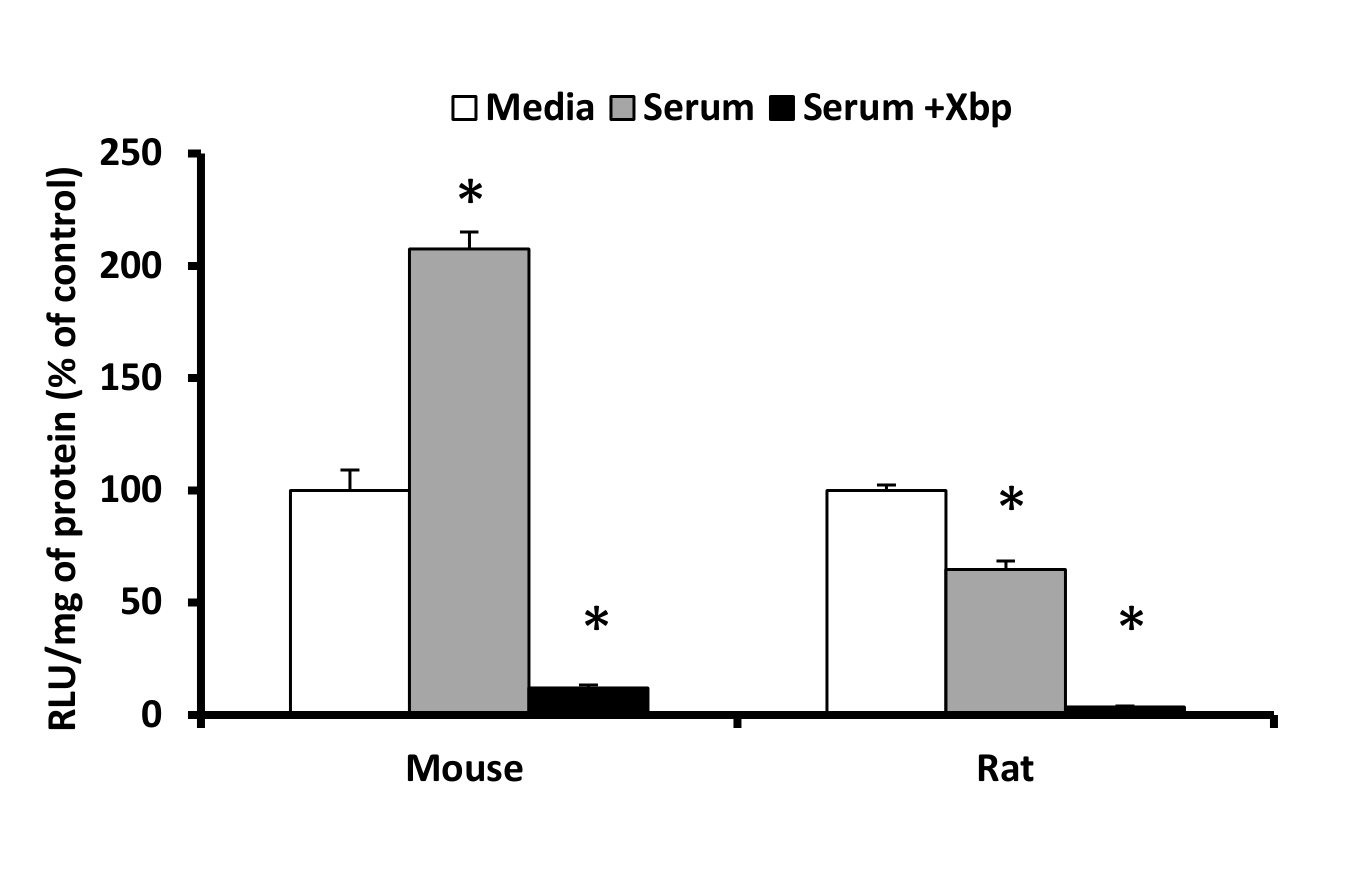

Supplement: S1 Fig — (A) Ad5 (2x10^10^ vp/mL) was incubated with RPMI-1640 media, 90% C57BL/6 mouse or Wistar rat serum in the absence or presence of 40 μg/mL X-bp for 30 min at 37°C. Virus suspensions were diluted 200-fold in serum-free media and 100 μL added to SKOV3 cells for 2 h at 37°C and then replaced with RPMI-1640 media with 2% FCS. Transgene expression was quantified ∼16 h post-transduction and relative light units (RLU) normalized to mg total protein. *p<0.001 vs. species matched media control. (TIF) [file ppat.1004673.s001.tif]

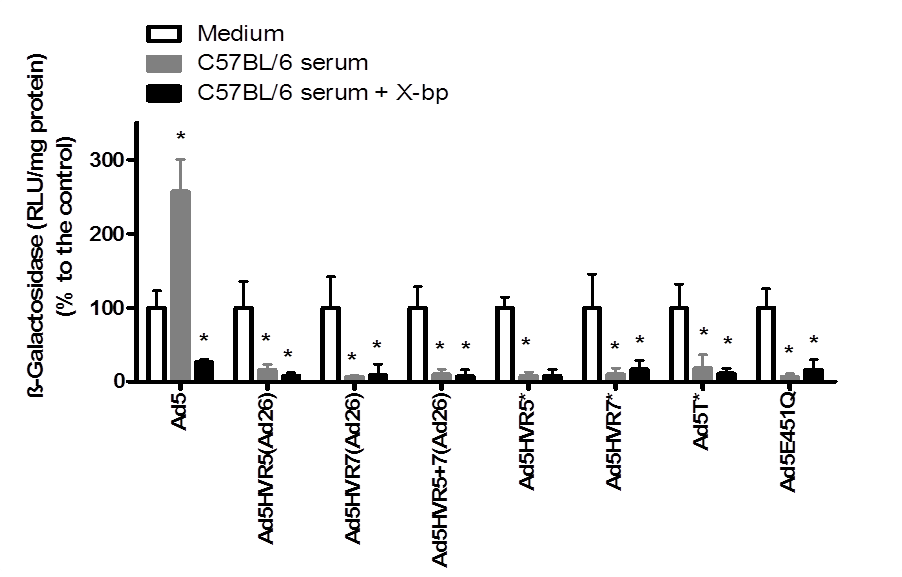

Supplement: S2 Fig — (A) Ad5 and the FX-binding deficient derivatives (2x10^10^ vp/mL of each vector) were incubated with RPMI-1640 media or 90% C57BL/6 mouse serum in the absence or presence of 40 μg/mL X-bp, for 30 min at 37°C. Virus suspensions were diluted 200-fold in serum-free media and 100 μL added to SKOV3 cells for 2 h at 37°C before being replaced with RPMI-1640 media with 2% FCS. Transgene expression was quantified ∼16 h post-transduction and relative light units (RLU) normalized to mg total protein. Graphs show transduction as a percentage of control (Ad transduction with serum free media alone). *p<0.001 vs. matched the control. (TIF) [file ppat.1004673.s002.tif]

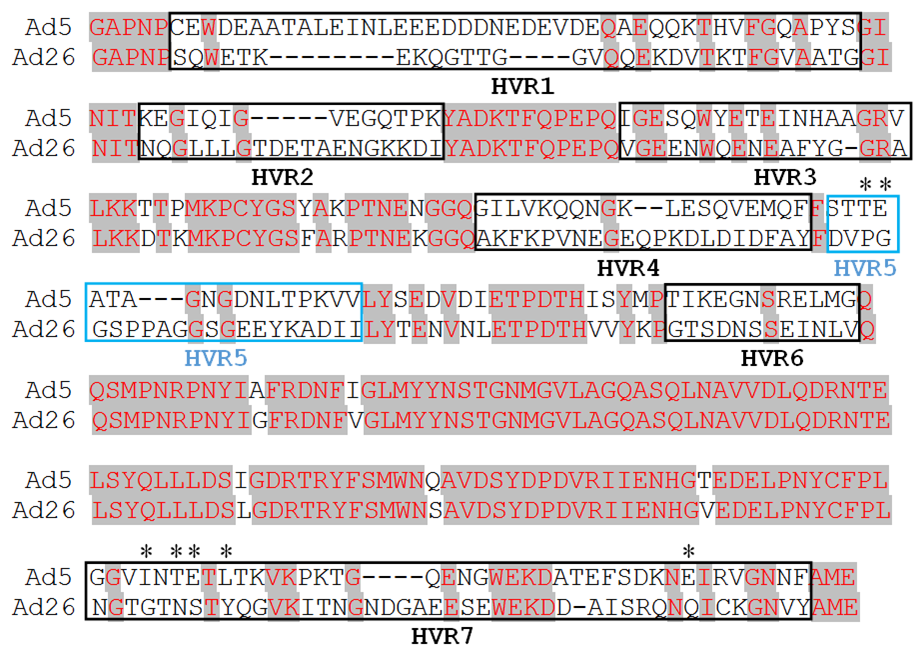

Supplement: S3 Fig — Amino acid sequence alignment of the Ad5 and Ad26 hexon HVR regions to highlight domains (HVR1, HVR2, HVR3, HVR4, HVR6 and HVR7, black box; HVR5, two blue boxes) and amino acids targeted for mutagenesis studies (point mutations highlighted by *). Ad26.HVR5C = Ad26 vector in which the Ad26 HVR1–3 and 5–7 were swapped with that of Ad5. (TIF) [file ppat.1004673.s003.tif]

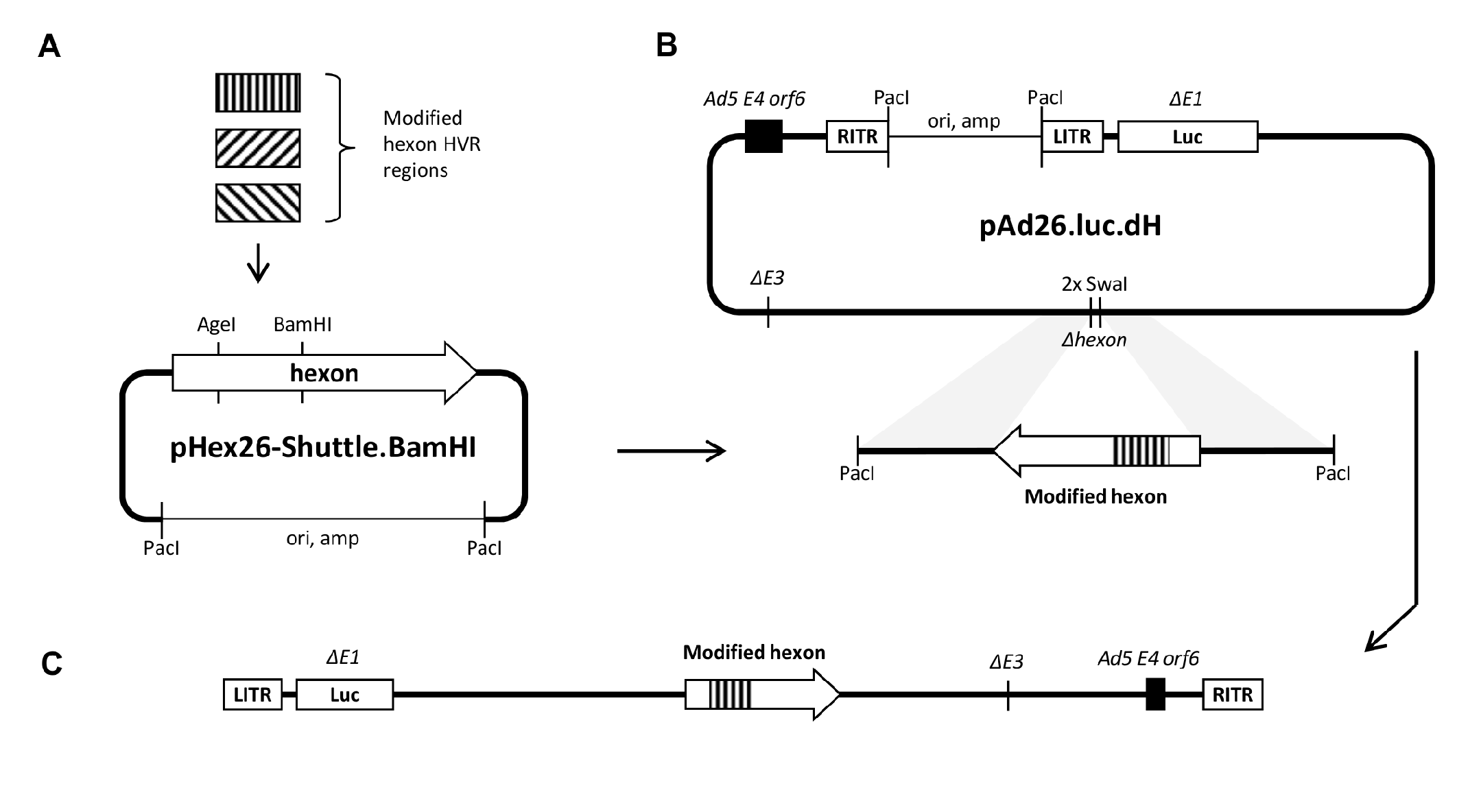

Supplement: S4 Fig — (A) Hexon sequence fragments containing the desired hexon modifications were gene synthesized and subcloned, as AgeI-BamHI restriction fragments, in place of the corresponding fragment within the ‘hexon shuttle’ plasmid pHex26-Shuttle.BamHI. This plasmid carries, between two PacI sites, a 6362-bp Ad26 genomic segment (corresponding to nucleotides 15755 to 22116 of genbank accession number EF153474) encompassing the hexon coding sequence as well as left and right flanking sequences of 2 and 1.5 Kbp, respectively. The unique BamHI site present in the hexon coding sequence within this plasmid had been introduced by a silent mutation of a cytosine to thymine at a position corresponding to position 19365 in EF153474. (B) The hexon modifications were subsequently introduced into the Ad26 vector genome by homologous recombination (in E.coli) between PacI-digested hexon shuttle plasmids (containing the desired modifications) and SwaI-digested pAd26.luc.dH. This latter plasmid is a derivative of pAd26.luc that carries, between two PacI sites, an Ad26.luc vector genome whose hexon gene is replaced by SwaI sites. (C) Hexon-modified Ad26 vectors were finally rescued by digestion of the hexon-modified vector genome plasmids by PacI and transfection of the resultant digestion products into E1-complementing cells. (TIF) [file ppat.1004673.s004.tif]

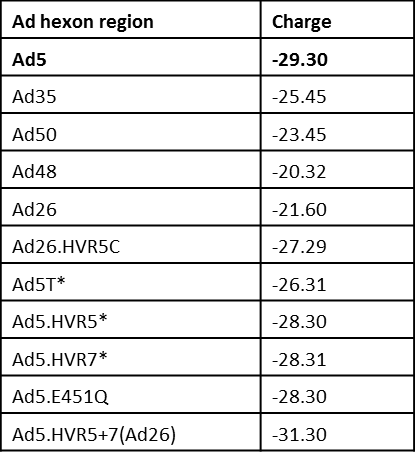

Supplement: S1 Table — Partial hexon amino acid sequences including the HVRs of Ad5, Ad50, Ad35, Ad26, Ad48 and chimeric vectors (see S3 Fig. for Ad5 reference sequence) were aligned. The net protein charges at pH 7.0 were calculated using CLC Genomics Workbench 6.0.5. protein analysis software. (TIF) [file ppat.1004673.s005.tif]

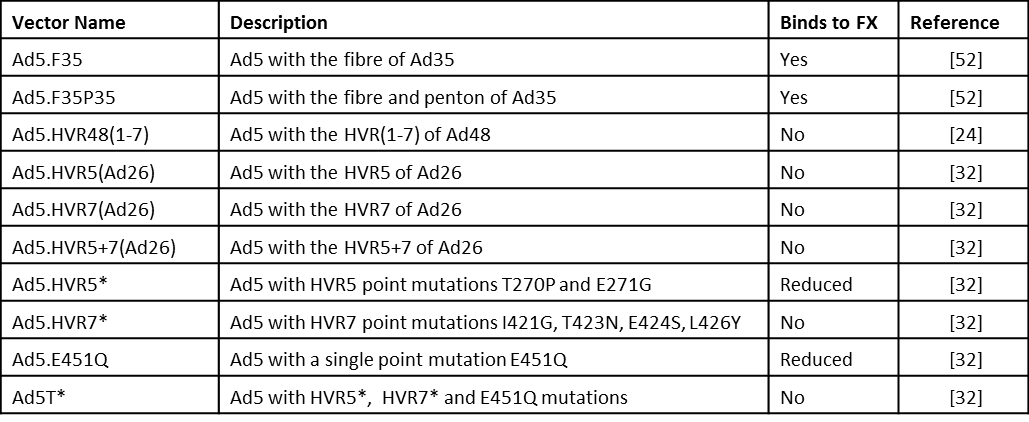

Supplement: S2 Table — Description of the regions swapped in the chimeric vectors and their ability to bind to FX. (TIF) [file ppat.1004673.s006.tif]

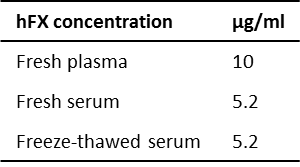

Supplement: S3 Table — An ELISA was used to measure the concentration of FX in human plasma and serum samples. (TIF) [file ppat.1004673.s007.tif]

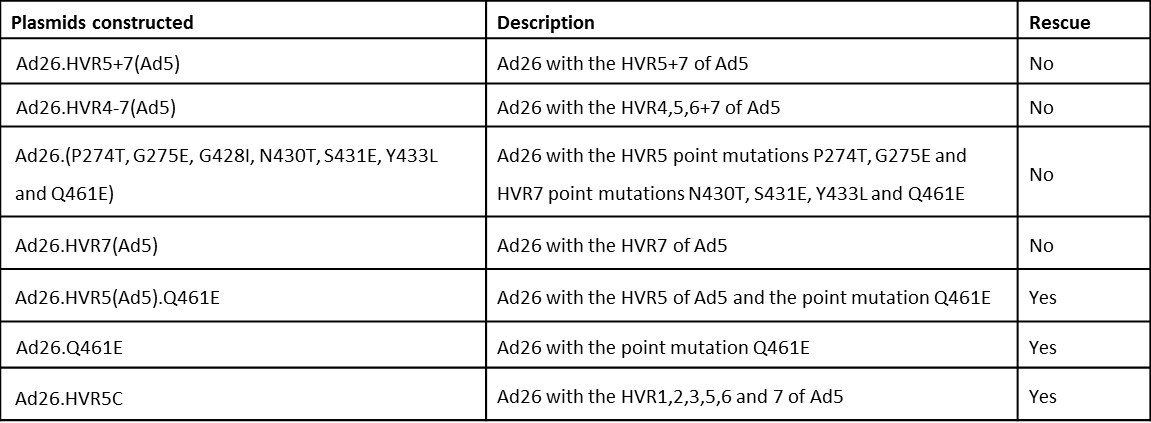

Supplement: S4 Table — Summary of all Ad26-based plasmid systems constructed and the ability to rescue virus particles in PER.C6/55K cells. (TIF) [file ppat.1004673.s008.tif]
